# Factors influencing nursing students’ learning in a clinical skills laboratory

**DOI:** 10.4102/hsag.v29i0.2631

**Published:** 2024-10-10

**Authors:** Catherine M. Hoffman, Juliana Willemse

**Affiliations:** 1 School of Nursing, Faculty of Community and Health Sciences, University of the Western Cape, Cape Town, South Africa

**Keywords:** clinical skills laboratories, learning, nursing, perceptions simulation, students

## Abstract

**Background:**

Achieving competence in clinical skills requires various resources and preparation time for undergraduate nursing students. Intentional opportunities should be created for student nurses to experience meaningful encounters in the clinical skills laboratory (CSL) to broaden their knowledge and competence. Therefore, factors that influence the competence in clinical learning in a CSL were explored.

**Aim:**

This study aimed to explore and describe the perceptions of nursing students regarding factors influencing their learning in a CSL.

**Setting:**

The study was conducted at a School of Nursing at a university in the Western Cape province.

**Methods:**

A qualitative descriptive design was adopted. Purposive sampling was used to select participants for this study based on specific inclusion and exclusion criteria. Following the attainment of ethical clearance, 10 focus group interviews were undertaken to explore the perceptions of nursing students regarding factors influencing their learning in a CSL. The focus group sessions continued until data saturation was established. Data were analysed using a thematic analysis process.

**Results:**

Three major themes emerged from the data analysis that was explored to make the following recommendations: physical environment, psychosocial environment and organisational environment.

**Conclusion:**

The results of this study highlighted the necessity for implementing interventions to enhance specific elements within the CSL, aiming to foster high-quality clinical learning experiences for nursing students.

**Contribution:**

Areas were identified within the CSL environment that requires enhancement to ensure the provision of quality clinical learning, thereby enabling student nurses to attain clinical competence.

## Introduction

Effective clinical teaching, developmental training and assessment of undergraduate nursing students can enable competent and self-assured professional nurses (Donough 2023). However, various studies indicate that nursing graduates experience a sense of being ill-equipped in terms of performing the required clinical skills in the real-world context (Vichittragoonthavon et al. 2020). It is expected of qualified registered nurses to display clinical competence of fundamental skills ensuring safe nursing practice. Because of limited opportunities for clinical skills learning at clinical placement facilities during undergraduate nursing training, clinical skills laboratories (CSLs) have become an essential component of nursing education (Msosa, Bruce & Crouch 2022).

Most educational institutions that offer qualifications in health-related professions make use of CSLs. These CSLs resemble real clinical facilities in which students are able to acquire clinical skills through simulation exercises (Jeppesen 2017). In preparation for the clinical experience, students are taught and are able to practice, the required nursing skills in the CSL under the supervision of a clinical supervisor (Thurling, Muthathi & Armstrong 2017). In a study conducted by Rahim (2018) in South Africa, the author believes that the principal objective of a CSL is to provide a safe environment for students to learn and practise new clinical skills before they start working on actual patients within a clinical setting. Students must be adequately prepared to carry out clinical skills competently and efficiently. Promoting theory–practice integration, educators and clinical supervisors are expected to facilitate students to improve their cognitive, psychomotor and affective skills (Hoffman & Daniels 2020).

Clinical competence is obtained through simulated, real-life experiences and repetitive learning opportunities (Fukada 2018). In the initial week of orientation at the School of Nursing (SoN), where this study took place, students are introduced to the skills laboratory methodology (SLM) according to Hoffman and Daniels (2020). The SLM consists of five phases, and its application is outlined in Table 1.

**TABLE 1:** Phases of the skills laboratory method.

Phases	Key concepts	Goals
Orientation	To focus on the rationale of the method	To gain insight into the SLM and various concepts
Visualisation	To form an image	To gain insight into nursing actions
Guided practice	Provide critical information Opportunity to practise skills on simulated patients Clarification of concepts Feedback	Execute actions under direct supervision
Independent practice	Self-directed learning	Execute actions independently
Assessment	Demonstrate competence in clinical skills Feedback	Guidance towards mastery of these skills

*Source:* Hoffman, C., 2023, *Perceptions of nursing students regarding factor influencing their learning in a clinical skills laboratory at a school of nursing in the Western Cape*, viewed 12 January 2024, from https://uwcscholar.uwc.ac.za/items/da348a17-bfad-45f0-b06d-cf967598096e/full

SLM, skills laboratory methodology.

There are four CSLs at the SoN where the study was conducted that are being used for clinical simulation. The focus is on the visualisation of clinical skills and facilitation of guided and independent practice sessions prior to assessment to establish clinical competence. Simulators that vary between low, medium and high fidelity are used in clinical teaching sessions. The provision of adequate resources to allow the repetition of clinical skills within an authentic CSL environment is important to improve competency in the clinical skills of students (Laari & Dube 2020). Being able to maximise a nursing student’s exposure to the CSL, the researcher explored the factors that could have an influence on them accomplishing competence in clinical learning.

### Purpose of the study

The purpose of the study was to explore and describe the perceptions of nursing students regarding the physical, psychosocial and organisational environmental factors that influence their learning in a CSL.

## Research methods and design

The researcher utilised an exploratory, descriptive design with a qualitative approach to explore the perceptions of nursing students regarding factors influencing their learning in a CSL. The exploratory and descriptive design explored the unknown territory and described the many aspects of the phenomenon under study (Dudovskiy 2018).

### Research setting

The study was conducted at a SoN in the Western Cape that forms part of a Community and Health Sciences Faculty at a university in the Western Cape province. In this setting, two SANC Regulatory Bachelor of Nursing programmes were offered in 2022 at the time of the study, namely Regulation 174 (R174) and Regulation 425 (R425). The R174 programme is a new programme offered since 2020 that replaces the R425 programme. The R425 was a legacy nursing qualification that is being phased out in line with the requirements of the Higher Education Qualifications Sub-Framework as published in the Government Gazette by the Minister of Higher Education, Science and Innovation in July 2016.

### Population and sampling

A purposive method of sampling was utilised to guide the selection of participants that would maximise the collection of rich data (Polit & Beck 2017). The accessible population was the total sample of all student nurses registered in 2022 for their first, second, third or fourth year of study. The population consisted of *N* = 693 undergraduate nursing students in the 4-year undergraduate programme at the identified SoN. All first to fourth-year students registered for a Bachelor of Nursing programme at a SoN in the Western Cape were invited to voluntarily participate in the study upon receipt of ethical clearance obtained from the Humanities and Social Sciences Research Ethics Committee of the university (HS20/10/58). Participants had to be in their first enrolment in the current year level to be included in the study in either the R174 or R425 undergraduate nursing programme. A total of 10 focus group interviews were conducted consisting of six to seven participants per group. The number of participants per group was determined by the availability of participants when focus group interviews were conducted. Data saturation was reached by the end of the 10th focus group interview, and there was no new information that emerged thus the total sample size was 54 participants (Table 2).

**TABLE 2:** Sample size summary.

Year level	Focus group interviews	Sample size	Participants per focus group
Year level one	3	20	7 students 7 students 6 students
Year level two	2	12	6 students 6 students
Year level three	3	19	7 students 6 students 6 students
Year level four	2	12	6 students 6 students

*Source:* Hoffman, C., 2023, *Perceptions of nursing students regarding factor influencing their learning in a clinical skills laboratory at a school of nursing in the Western Cape*, viewed 12 January 2024, from https://uwcscholar.uwc.ac.za/items/da348a17-bfad-45f0-b06d-cf967598096e/full

### Data collection

Focus group interviews were selected as a data collection method because it allowed the researcher to pose open-ended questions, during which the views and opinions of the participants were obtained to gain an understanding of their experiences (Creswell & Creswell 2018). An interview schedule was prepared in advance of the focus group interviews and was consistently utilised with all focus group interviews (Creswell & Creswell 2018). The development of the interview schedule was influenced by the research question: ‘What are your perceptions on the factors influencing your learning in a CSL as an undergraduate?’ The three main factors of the theoretical framework for a clinical learning environment (Haraldseid, Friberg & Aase 2015) were applied to this study to explore how the physical, psychosocial and organisational environment influence the learning of nursing students in a CSL (Table 3). The framework identifies three main factors for a clinical environment. Physical environment: physical equipment, facilities and standardised procedures are sub-contexts of the physical environment (Haraldseid et al. 2015). Psychosocial environment: psychosocial refers to the close association between psychosocial aspects of our experiences (Haraldseid et al. 2015). Organisational environment: entails the course structure and institutional resources (Haraldseid et al. 2015). The theoretical framework and its various components, with the objective, influenced the development of the nine questions in the interview schedule. Probes were used to prompt the participants to supply richer, in-depth information (Gray, Grove & Sutherland 2016). Interviews were conducted in the research setting in a suitable venue. Privacy was ensured by using a soundproof, well-ventilated room with a door that was closed, which encouraged an open and relaxed environment. The interviews were audio recorded with written permission from the participants and lasted approximately 50 min. A printed information sheet was given to the participants with the relevant information regarding the study. Written consent was obtained from the participants after reading the information sheet. Participants were informed that participation was voluntary and that they could withdraw from the study at any stage without any negative impact on their studies. The focus group interviews continued until data saturation was reached where no new data emerged (Gray et al. 2016).

**TABLE 3:** Focus group interview questions.

Questions	Probes to guide the researcher
**Physical environment**	
To what extent do you perceive the clinical skills laboratory to be equipped with equipment for your learning needs in your current year level?	Explain more, elaborate and give examples Manikins, equipment and simulation manikins
To what extent do you perceive the clinical skills lab accessible for practice purposes and self-directed learning?	How did you experience the booking schedules?
To what extent do you perceive the learning tools in the clinical skills lab to be accessible?	Explain more about the videotapes, clinical consultations with the clinical facilitator and procedure guidelines
How do you perceive the standardisation of clinical procedures?	How to perform a clinical skill? Standard guidelines
**Organisational environment**	
How do you perceive the department’s human resources interaction with the nursing student in the clinical skills laboratory at your year level?	Clinical supervisor, simulated patients, clinical skills laboratory coordinator and clinical coordinator
What are the challenges you perceive within an organisation and its relation to the clinical skills laboratory?	Environment, training session group numbers per station and short training sessions
**Psychosocial environment**	
What are your expectations for a clinical training session in the clinical skills laboratory?	Time, feedback, practice and facilitation
How do you perceive the feedback given during clinical training sessions?	Positive, constructive, objective
What is your perception of the support provided by the department to the student within the clinical skills laboratory?	Additional skills training sessions

*Source:* Hoffman, C., 2023, *Perceptions of nursing students regarding factor influencing their learning in a clinical skills laboratory at a school of nursing in the Western Cape*, viewed 12 January 2024, from https://uwcscholar.uwc.ac.za/items/da348a17-bfad-45f0-b06d-cf967598096e/full

### Data analysis

Data analysis is a continuing process in the research study (Creswell & Creswell 2018). The researcher utilised thematic analysis, which is a systematic method for qualitative data analysis. Recordings from the focus group interviews were transcribed verbatim to enhance trustworthiness. The six-step approach of Braun and Clarke (2022) was used to analyse the data: Step 1: become familiar with the data; Step 2: generate initial codes; Step 3: search for themes; Step 4: review themes; Step 5: define themes and Step 6: write-up the data analysis. The theoretical framework for a clinical learning environment by Haraldseid et al. (2015) guided Step 2 and Step 3 to generate initial codes and themes, with the physical, psychosocial and organisational environment of a CSL. Themes were reviewed in consultation with the supervisor and an independent coder to cross-check initial codes and to ensure the trustworthiness of the findings.

A thematic map was created of the data to define themes. In conclusion, in the manual data analysis following the six steps of Braun and Clarke (2022), the researcher used the Atlas ti version 22 research software program to generate codes to support the findings. This was carried out by organising the data, bracketing and writing a word representing the category. The independent coder followed the same format, using Atlas ti version 22.

### Measures of trustworthiness

Trustworthiness refers to the quality of a research study and whether the findings and interpretations can be trusted (Lincoln & Guba 2016). Trustworthiness in this study was applied in terms of credibility, reflexivity, confirmability, dependability and transferability. Credibility refers to establishing truthfulness in the findings and interpretations of the research study (Lincoln & Guba 2016). The truthfulness of the findings was judged by the participants and the supervisor of the research study (Korstjens & Moser 2018). Credibility was ensured by recording the focus group interviews and transcribing them verbatim. The transcripts were returned to the participants for them to read and then share their interpretation of the findings. *Dependability* refers to the consistency of the data over a period and the conditions of the study (Polit & Beck 2017). The supervisor reviewed the inquiry process to check for consistency (Lincoln & Guba 2016). *Confirmability* is the degree to which the findings of the research study can be validated by other researchers (Korstjens & Moser 2018). The research supervisor and an independent coder cross-checked the findings. Confirmability was further established with direct quotes from participants, which were included in the findings to provide a rich description of the findings to ascertain that the data represent the information of the participants (Polit & Beck 2017). *Transferability* refers to the degree to which the results of qualitative research can be transferred to other contexts or settings (Korstjens & Moser 2018). The researcher ensured transferability by providing a detailed description of the research setting, the participants and the methods utilised for data collection and data analysis. *Reflexivity* is the process of self-reflection for the researcher to ensure that bias, preferences and preconceptions are noted. The researcher was mindful of her own preconceived opinions about the phenomenon under study (Polit & Beck 2017).

### Ethical considerations

The code of ethical rules and principles is drafted by the professional associations that govern scholarly research in the various disciplines (Creswell & Creswell 2018). Ethical clearance was obtained from the Human and Social Sciences Research Ethics Committee (HSSREC reference number: HS20/10/58) at the University where the study was conducted. Permission to conduct the proposed study was obtained from the relevant university structures to ensure the upholding of high ethical standards in this research process. The researcher adhered to the following to ensure the protection of the identity and rights of the participants. Each participant received an information sheet explaining the purpose of the study. Focus group interviews did not include any information about the personal nature of the participants, and pseudonyms were assigned to each participant and included on the transcripts. Participants were informed both verbally, and on the written consent form, that all collected data will only be utilised for research purposes and recordings, and all other documentation will be destroyed after 5 years. All recordings and transcripts were code-encrypted.

## Findings and discussion

Three major themes and nine sub-contexts emerged from the study exploring the perceptions of nursing students regarding factors influencing their learning in a CSL. A synopsis of the theoretical framework aligned with the identified themes and sub-context is presented as outlined in Table 4.

**TABLE 4:** Theoretical framework aligned with themes and sub-contexts.

Theoretical framework	Themes	Sub-contexts
**Physical environment sub-context:**	
Equipment, manikins, simulation manikins, accessibility, booking schedules Learning tools: Videotapes, consultations with clinical supervisors, procedure guidelines, standardisation of clinical procedures	1. Factors of the physical environment of the clinical skills laboratory influencing the learning of nursing students	1.1 Clinical skills laboratory is well equipped and mimics the hospital environment 1.2 Challenges with equipment that hamper procedure. 1.3 Accessibility and operation of medium and high-fidelity mannequins for simulation 1.4 Limited accessibility for guided practice and self-directed learning 1.5 Difficulties with learning tools 1.6 Standardisation of clinical procedures
**Organisational environment sub-context:**		
Department human resources: Clinical supervisors, simulated patients, clinical skills laboratory coordinator, clinical coordinator Challenges: Training sessions group numbers per station, length of training sessions	2. Factors of the organisational environment influencing the learning of nursing students	2.1 Challenges with human resources utilised for clinical skills laboratory activities
**Psychological environment sub-context:**		
Expectations: Time, feedback, practice and facilitation	3. Factors of the psychosocial environment influencing the learning of nursing students	3.1 Expectations of the nursing students regarding their experience of the clinical skills laboratory 3.2 Support provisions for nursing students to meet their learning needs

*Source:* Hoffman, C., 2023, *Perceptions of nursing students regarding factor influencing their learning in a clinical skills laboratory at a school of nursing in the Western Cape*, viewed 12 January 2024, from https://uwcscholar.uwc.ac.za/items/da348a17-bfad-45f0-b06d-cf967598096e/full

### Theme 1: Factors of the physical environment of the clinical skills laboratory influencing the learning of nursing students

Six sub-contexts are associated with theme one, which focusses on the factors of the physical environment and their influence on student learning. Participants noted that while the CSL is well equipped in some respects, there is a deficiency in necessary equipment or existing equipment is faulty, negatively affecting their learning requirements. The findings suggest that nursing students face restricted access to the CSL and require more sessions to meet their learning needs and achieve necessary competency. Additionally, students identified challenges in using outdated clinical learning tools, procedures and demonstrations, leading to confusion about what is utilised in real clinical placement facilities. This discrepancy in appropriate and updated resources within CSLs may impact the quality of clinical learning, which could result in the delivery of substandard nursing practice (Mbakaya et al. 2020).

#### Sub-context 1.1: Clinical skills laboratory is well equipped and mimics the hospital environment

Participants conveyed that the CSL is adequately equipped and replicates the ambience of a hospital setting. Ensuring the effectiveness of simulation is crucial for the simulation environment to closely imitate real-life scenarios encountered in clinical settings (Saifan et al. 2021). Clinical skills laboratories contribute to creating a positive learning environment, fostering the students’ learning process (Jeppesen, Christiansen & Frederiksen 2017). Moreover, participants expressed that the well-equipped CSL allowed them to transfer their skills from the laboratory to the hospital setting. Arkan, Ordin and Yılmaz (2018) suggest that every Higher Educational Institution (HEI) offering an undergraduate nursing programme should strive to bridge the gap between theoretical knowledge and clinical practice. Participants reported the following:

‘It’s very modern and it’s really wonderful because it gives us as close to a real patient working with a patient. It’s very true to life. So, it give [*sic*] us a real idea of what we can expect with different patient.’ (Participant 3, FG1, BN1)

‘We have real life mannequins that we can practise on … This is basically like a hospital. So, everything that we need in the hospital, we need to learn to do in the hospital we have here and we can practise.’ (Participant 2, FG3, BN1)

‘… a lot of things we do in skills that we also get the chance to do in hospital.’ (Participant 1, FG3, BN1)

‘I think the skills lab is well equipped because most of the stuff that we use in our clinics, they are available here. We like to look at beds also like she said earlier also we look at the vital signs machines we also use. We also use that at the hospital, which just makes it more easy and more easy doing the things …’ (Participant 8, FG3, BN1)

Therefore, it is essential that the CSL is authentic to help minimise the reality shock that the world of clinical practice can evoke and to ensure students are adequately prepared for the reality of the hospital environment and working with actual patients (Laari & Dube 2020).

#### Sub-context 1.2: Challenges with equipment that hamper procedures

One of the main concerns identified by participants was accessibility to the necessary equipment in the CSL to practise certain procedures. This concern was confirmed by the research of Moyimane, Matlala and Kekana (2017) where nursing students were adversely impacted by physical shortages such as lack of equipment. Some participants reported on the negative impact that faulty equipment had on their ability to practise their clinical skills within the CSL. A participant remarked:

‘Some of the things are broken, so it’s really hard for us to practise blood pressure in the skills because we have to swap and change the whole time.’ (Participant 3, FG1, BN1)

Students in a study conducted by Thurling et al. (2017) also had to improvise with procedures because of the lack of clinical equipment and other resources, which affected the quality of their clinical learning. Participants remarked on the use of mannequins that were faulty:

‘… all the mannequins have something wrong with them. Either the head is loose or the arm. So even if you would have washed, a practise in washing … the patient, it will be difficult because the legs will be coming of. But if you want to lift it up or practise passive exercises as to demonstrate, it would be really difficult.’ (Participant 3, FG1, BN1)

Participants further remarked on the lack of equipment available to practise. Adequate equipment should be available to create a realistic environment in the CSL to ensure the quality of nursing skills performance (Labrague et al. 2019):

‘… groups working in the skills lab and practising the same skill, the equipment might not be enough for everyone to go around. So, there might be three babies. Everyone wants to do baby washing practise, but then a group is left out because there’s not enough equipment.’ (Participant 5, FG1, BN1)

‘… just some of the stuff. We don’t have or it doesn’t work. Like with suturing. There is no suture like we need to bring our own or with practicing certain stuff and the stethoscopes are broken.’ (Participant 2, FG6, BN3)

Msosa et al. (2022) reiterate that CSLs should be better equipped to ensure student learning. Therefore, the institution must ensure that the CSL is effectively equipped with the required equipment.

#### Sub-context 1.3: Accessibility and operation of medium and high-fidelity mannequins for simulation

Higher Educational institutions utilise computerised high-fidelity simulators to facilitate clinical learning. They are able to mimic real-life scenarios in the CSLs to facilitate the student’s ability to transition from the CSL to the hospital environment (Mothiba, Bopape & Mbombi 2020). Participants reported that there were limited opportunities for exposure to medium- and high-fidelity mannequins because of either equipment not functioning or staff being uncertain regarding how to operate the mannequins. A participant commented:

‘… she was quite strict. We were just busy by the mannequin … she saw us and she was going on … And then we may not have pens or pencils also nearby the mannequin, which is understandable, but it was just overreaction and being too protective over the mannequins and not allowing the first year to get to know certain things … get too comfortable with the mannequin and doing certain things, especially because it’s our first time. So maybe too protective because I know we’ve never been able to use the simulated mannequins at all.’ (Participant 3, FG3, BN1)

Clinical staff working in the CSL must be effective in operating these mannequins in order to ensure students benefit optimally during their learning process. Facilitators of simulation learning interact in the environment and guide the session (Hanshaw & Dickerson 2020).

‘… what I have noticed is that even those that are working are not working properly and some other supervisors try to use them. I think maybe some of them are not orientated enough on how to use them …’ (Participant 7, FG7, BN3)

Participants in their third year were exposed to the medium- and high-fidelity mannequins, but students in their first year had limited exposure because of the scope of clinical skills that can be demonstrated with these mannequins. Although students had limited exposure to high-fidelity mannequins, they recognised high-fidelity simulation as a valuable preparation for clinical practice (Bowen-Withington et al. 2020).

A participant in the fourth year recalled an encounter in the third year with these mannequins:

‘Primary Health Care we had to do the jaundice, the phototherapy. Like, that was very, almost like a real-life baby. But it was very precise of how we did it in the clinic. So, I able to relate what we did in the skills lab to what we had to in the hospital.’ (Participant 2, FG10, BN4)

Resource constraints, such as outdated high-fidelity teaching models that can simulate a clinical scenario, can interfere with the students’ acquisition of clinical skills (Msosa 2017):

‘Sometimes the mannequins won’t work … now the abdominal wouldn’t work.’ (Participant 2, FG7, BN3)

#### Sub-context 1.4: Limited accessibility for guided practice and self-directed learning

A student nurse needs adequate knowledge, preparation and demonstration of safe nursing practice to ensure that they develop their core competencies to ensure safe clinical practice (Kerr et al. 2020). Participants across the 4-year levels have expressed how difficult it was for them to gain access to the CSL for self-directed learning sessions. This was because of the limited spaces available to prevent overcrowding in the skills labs during these sessions as indicated by participants:

‘… I would say there’s not enough because there’s a set amount of people that are allowed to be in the skills lab … it’s very difficult for me to book a place in for next Friday because it will always be full … the same people constantly book, because I understand they want to now all fill their hours. But now then there’s other students that are left with nothing. So, I would say it’s not accessible because there’s only a small amount of people that can go into the SDL.’ (Participant 3, FG2, BN1)

‘… there’s only a limited amount of spaces open to be booked.’ (Participant 4, FG1, BN1)

‘We want to improve on our skills, but we don’t get the chance because it’s fully booked.’ (Participant 6, FG1, BN1)

Participants across the 4-year levels further elaborated on the impact that the limited access to the CSL had on their clinical learning process, during self-directed learning, being left with no opportunity to practice led to extending the time it took for them to become competent in a clinical skill. In a study by Msosa et al. (2022), students viewed access to simulation as the most appropriate way of transitioning theory content into clinical practice and access to the CSL subsequently allows the building of confidence and gaining competence. Participants reported their challenges with access to the skills laboratory:

‘We only get self-directed learning for one day, which is a few hours. Yes, and then you got to do everything in a minute in a hurry, meaning that you’re not learning quite well. Like we should.’ (Participant 1, FG6, BN3)

‘We don’t have time for self-directed learning … one of the issues and other thing is sometimes there is not space for everyone. So, some of us come from so we get the skills lab then it’s full so we do have to go back.’ (Participant 5, FG4, BN2)

Practising skills in a CSL is essential in order for the student to develop and refine their clinical skills and allowing them to make mistakes without harming a patient, there is an opportunity to rectify mistakes safely in the CSL. Swift, Henderson and Wu (2022) place emphasis on simulated nursing as a means for nursing students to develop self-confidence in psychomotor clinical skills.

#### Sub-context 1.5: Difficulties with learning tools

Clinical facilitators were not always available in CSL to guide the students as they needed, because of other clinical obligations. However, demonstration videos on the various clinical skills relating to the clinical competencies that have to be achieved were available for each year level to view. The availability of the skills demonstration videos could provide the students with opportunities to repeatedly review their clinical skills at their convenience and to practise the clinical skills should the clinical facilitator not be available (Chuang et al. 2018). Participants expressed challenges with discrepancies with regard to the learning tools. Some participants noted that the information included in the procedure guide did not reflect the actual steps for the procedure on the video, as pointed out by the following participant:

‘We got orientation say now for jaundice and lymph nodes. We got orientation on that and that is according to the module, which is the tool that we learn from. Then we done it in the assessment and clinical placements. So, within the module guide there will be maybe a lymph node that is missing. And the way they describe the landmarks is different to the way the supervisors wants us to memorise it and how we should memorise it … So personally, when I done my assessment in the clinic, I got the landmarks wrong for the lymph nodes because I studied according to what they provided us in the module guide.’ (Participant 2, FG7, BN3)

Additionally, participants felt that the procedure guide was inconsistent in relation to the actual procedure they were expected to complete. According to Swift et al. (2022), students prepare for their CSL sessions by reviewing pre-reading material prior to the session in order to have the necessary pre-requisite knowledge. A participant commented on the following:

‘I think that for the most part, how module guides have been very inconsistent. Like, for example, we’ll still have stuff that’s like in the printed version that’ll say to be clarified … procedures are completely different or it doesn’t outline it well enough in the module guide as we are being assessed on … it’s supposed to be a guide and it’s ultimately not.’ (Participant 4, FG5, BN2)

The omission of procedures from the clinical module guide had a negative impact as students needed guidance on how to perform a procedure, for example how to perform the last offices or how to carbonise a bed, as stated below:

‘… concerning the guidebook, there was one procedure I couldn’t quite find the one about the last offices and the carbonising of the bed. So, I was upset about that …’ (Participant 6, FG2, BN1)

Participants reported that although the clinical skills-related videos were available on the e-learning platform, the videos were outdated and in certain aspects, not the current practice. The advantages of audio and video recordings in higher education enhance student-learning outcomes and reduce the anxiousness experienced by nursing students (Tohidi et al. 2019). A participant reported the following:

‘I also think it’s outdated because sometimes the equipment in that videos is equipment we’re not using anymore.’ (Participant 1, FG4, BN2)

Procedure guides should be consistent and accurate so that the student can revert as required to recall the steps of a clinical procedure. All CSL staff should be involved when developing study guides for skills training to ensure differences are eliminated (Msosa 2017).

#### Sub-context 1.6: Standardisation of clinical procedures

Participants found it quite worrying that procedures are demonstrated differently by clinical facilitators, with some of the participants stating they feel stressed because of this. Participants stated the following:

‘I think every supervisor is great at what they do, but everyone has a different way of doing things. So sometimes if we have visualisation, the one week we do it a certain way and the next week we’ll have a guided practise with a different supervisor and we would be confused on certain things because they just do it differently.’ (Participant 1, FG1, BN1)

‘… some students would say, yes, the facilitator explained this, but some have no idea what’s going on. I feel sometimes the level of teaching is not the same and if you are unfortunate enough to get placed with the facilitator for a very important skill and you lack some of the information, I feel like it’s in our disadvantage …’ (Participant 2, FG2, BN1)

Additionally, the participants experienced a degree of stress because of the expectations from clinical facilitators. A participant had the following to say:

‘… then they kind of expect you to do what they expect … but then you weren’t there for the visualisation at that specific supervisor. So, it’s quite stressful.’ (Participant 5, FG1, BN1)

Furthermore, participants noted that the learning tools in conjunction with the procedure being demonstrated by the clinical supervisor did not correlate, and there was a skill variation among CSL staff members (Msosa 2017). Participants noted the following:

‘But most of the time then its three different resources that are all saying different things. So, you don’t know what is beneficial for the patient and you kind of have to use your own understanding which leaves room for error.’ (Participant 4, FG5, BN2)

‘It’s like when the supervisor explains the procedure and when you look at the guideline, sometimes it clashes. It’s not the same thing.’ (Participant 1, FG4, BN2)

‘I certainly feel that supervisors should get trained the same thing because it’s really frustrating because even when you do OSCE, so you do it a certain way that your supervisor told you. But then on that day you don’t get your supervisor, obviously, and then that marks you down because you’re not doing it according to the way they do it. So that is really unpleasant.’ (Participant 1, FG7, BN3)

Students’ ability to perform nursing skills is developed in the process of providing care in clinical practice, following the acquisition of the necessary knowledge and skills acquired in the CSL (Nakayoshi et al. 2021). The statements indicate that student learning has been impacted by the discrepancies experienced with clinical procedures, which influenced their competence in clinical procedures.

### Theme 2: Factors of the organisational environment influencing the learning of nursing students

Clinical nursing education should be delivered in an environment within an organisational culture that encourages learning and teaching activities for the student (Dube 2018). Organisational factors in nursing education comprise teaching schedules, rules governing the skills laboratory, quality and quantity of staff and the size of the CSLs (Msosa 2017).

#### Sub-context 2.1: Human resources utilised for clinical skills laboratory activities

Participants expressed various challenges they have experienced with human resources utilised within the CSL for practical purposes. In the process of facilitating the clinical learning process, clinical facilitators should endeavour at all times to work towards the common goal of safe and high-quality patient care (Bruce & Klopper 2017). A participant stated the following:

‘… when they reach my group, which is the last group, they (clinical supervisors) might not be as excited about the procedure or they are rushing everything along to get to the facilities, so we are not afforded the same quality as the first two groups.’ (Participant 5, FG2, BN1)

The quality of learning in the CSL regulates the ability of students to transition and prepare for practice (Msosa et al. 2022). Participants have stated that there are not enough clinical facilitators allocated for their clinical training sessions that impacted their learning; they were not able to receive the necessary feedback. Participants said the following:

‘So we probably have a big group with one supervisor and we’re not having too much information.’ (Participant 1, FG4, BN2)

‘… if it is a clinical skills [*sic*], I think it is supposed to be a small group of student, but now we have to be like more than 60 students … in one class by one clinical supervisor. So now it is a big class. We cannot focus. Everyone cannot get a chance to talk about one thing because now is like a lot of people, they can’t manage time.’ (Participant 2, FG9, BN4)

‘We only have one supervisor who will teach us. Maybe they will us to watch the video and write. Then that supervisor have [*sic*] to check for every student what they wrote.’ (Participant 5, FG10, BN4)

‘This is how it goes. We come to class for theory and then the same class. The whole amount is going to come for the skills in one class. So, for 2 hours, one supervisor or two. So, I think it’s too much for one person to deal with the whole class.’ (Participant 6, FG10, BN4)

A characteristic of a good clinical demonstration ensures that everyone attending the demonstration can see what the educator is demonstrating, and groups are typically small in order to facilitate interaction between the clinical facilitator and the students (Bruce & Klopper 2017). From the data collected, it was clear that there are challenges experienced in terms of booking for self-directed learning, the CSL programme and the time limitations of training sessions within the CSL. A participant stated the following:

‘… it’s always a hassle to actually get booked for SDL. I remember in the beginning of the year they told us we can book online, but I’ve tried so many times and the website doesn’t work.’ (Participant 2, FG2, BN1)

‘… the booking is the issue because there’s never the book outside.’ (Participant 1, FG4, BN2)

Participants stated that they had not been issued with a clinical schedule for their CSL activities to indicate what they needed to prepare for. A participant stated the following:

‘The other years they will give you a clinical skills schedule. So, when you come to skills, you know what to expect. You know what to prepare. But with fourth year, you never know what you’re getting. You come in, one group will do genogram and other group will do MSE. So, you don’t even know what you’re coming for, so you are not able to prepare for it.’ (Participant 3, FG10, BN4)

‘Compared to other years they used to give us and even module guides that is talking about which week are we doing this practise. We just get surprised that we are studying this topic. But they expect us to know the topic. So, we do not know. We get confused and it’s affecting our learning.’ (Participant 5, FG10, BN4)

Additionally, participants have stated the need for facilitation during their self-directed learning sessions as they feel lost and require some form of guidance to indicate that what they are practising is correct. Participants said the following:

‘There’s sometimes not even like a supervisor present like to guide is when we have SDL.’ (Participant 2, FG4, BN2)

‘I also think like there should be someone in the SDL like a facilitator who will be there for each station to help students, to help them train, because even though it’s self-directed learning, but sometimes you not sure of the thing you did …’ (Participant 4, FG2, BN1)

Self-directed learning in a CSL should be a reinforced practice with the assistance and insight of clinical facilitators (Kerr et al. 2020).

### Theme 3: Factors of the psychosocial environment influencing the learning of nursing students

The psychosocial environment identified the experiences of students in terms of their expectations when attending the CSL, as well as the support provided to them to assist them to meet their learning needs (Haraldseid et al. 2015).

#### Sub-context 3.1: Expectations of the nursing students regarding their experience of the clinical skills laboratory

Participants have expressed what they expect from a training session facilitated in the CSL. Participants expect to exit the CSL knowing they have learned valuable information, which could assist them in their learning process (Sheikhaboumasoudi et al. 2018).

‘I would like to leave the skills lab feeling like, you know, knowledgeable, like I’ve gotten enough information …’ (Participant 6, FG1, BN1)

‘I think my feedback in general is just I don’t want to leave being confused or unsure about something. If I leave a session, I like to know exactly how to do the procedure.’ (Participant 6, FG5, BN2)

Additionally, participants have expressed that they wanted to feel confident enough to practise after training sessions. Self-directed learning increases confidence and competence during clinical experiences (Kerr et al. 2020). Participants agreed:

‘I expect to leave feeling confident in the procedure that we were practising, and or at least even if I don’t feel competent, I’d like to leave feeling like almost encouraged that I will be able to do it with some extra practise.’ (Participant 4, FG8, BN3)

‘I feel like we do get feedback, but like they said, like, if they would make us practise more, then they would actually understand and see what everyone else in the group can improve on.’ (Participant 4, FG8, BN3)

Participants in their fourth year have expressed that they expect the same consistency from their first year up until their fourth year with regard to the CSL programmes (Dunbar 2018). A participant explains:

‘I’m expecting them in the school to carry on with the method they use from foundation one to the year of first year to third year when they dealing with the skills …’ (Participant 6, FG10, BN4)

Participants in their first year expressed that they expected clinical facilitators to be more understanding and patient with them because they are first-year students (Sweet & Broadbent 2017). First-year participants reported the following:

‘I just want the supervisor to be more invested in our learning … they should understand that we are only first year and that we still need to get the hang of things so they could be more understanding and be more patient and just allow for us to learn ….’ (Participant 5, FG2, BN1)

‘I remember that the group I was with, we were shouted at so badly. And I just remember, you know, thinking … is this how it’s going to be? You know? I remember feeling a lot of stress and I was nervous to practise that because, you know, they can influence your confidence in certain procedures. And I think it should be rather more of an encouraging environment and that we all there to learn.’ (Participant 3, FG1, BN1)

Facilitation of a student can strengthen the clinical facilitator–nursing student relationship to develop confidence and competence among nursing students (Gemuhay et al. 2019).

#### Sub-context 3.2: Support provisions for nursing students to meet their learning needs

Participants expressed that support is provided to them. It is important to note that effective support provided by clinical facilitators results in the improvement of clinical nursing education (Bhurtun et al. 2019). The clinical facilitators would make provisions wherever they could to assist, as communicated by the following participants:

‘I would say like I’ve been getting enough support with regards to procedures because whenever we do a procedure, they demonstrate to us then the access to the practise, then they do a guided practise, they give you a feedback … Then the supervisor explained to you, and you can always say, Ma’am, can I come again to do another guided practise?’ (Participant 3, FG4, BN2)

‘The supervisor is very helpful in skills lab and you need help, they always willing to help.’ (Participant 6, FG10, BN4)

‘If I have a question, if I’m finding something difficult to comprehend and then our supervisor always comes on a Monday for me, so he sticks to a set time so that you can reach all the students. So, he will take us through an hour and a half, teach us the assessment that he needs to teach us. And then after that, for like 30 minutes, we would have an open session. We would also ask questions, we would ask new questions and he would provide us with more information.’ (Participant 2, FG7, BN3)

A successful CSL needs to comprise an encouraging psychosocial environment team climate, active student learning experiences and supportive supervisory interaction (Ergezen, Akcan & Kol 2022). These factors may determine the achievement of clinical learning competence and promote the self-confidence of students.

### Limitations

The study was conducted with a sample from one SoN at a university in the Western Cape province, thus limiting the generalisation of the findings to other HEIs offering nursing training using CSLs.

### Recommendations

The findings indicate the need to implement interventions to improve certain aspects of the CSL to promote quality clinical learning for nursing students. Recommendations emerging from this study emphasise the need to address the following matters: faulty equipment, accessibility, outdated resources, standardisation and provision of human resources for clinical activities. Nursing students should have access to equipment that is functioning optimally to ensure the promotion of quality clinical learning to provide a more realistic experience when practising skills and procedures. Planning and scheduling should be reviewed to ensure that all year levels can be accommodated in the CSL for both guided practice and self-directed learning sessions. There should be an update of skills-related videos to ensure that they reflect current practice. High-quality standardised training should be offered to all clinical facilitators to ensure the standardisation of all clinical procedures for all year levels. Adequate human resources are required to ensure that the clinical facilitator-to-student ratio during CSL sessions is adequate and allows sufficient interaction with students, which will ensure quality in the clinical programme being offered.

## Conclusion

The study explored the perceptions of nursing students regarding factors influencing their learning in a CSL and the three themes emerged, which reflected the experiences of the nursing students. The physical CSL was well equipped and mimicked the real-life hospital environment. The experiences gained in the CSL assisted participants in their learning process in terms of developing competence in expected clinical learning tasks. However, challenges with equipment in the CSL hampered the performance of procedures and the ability of students to practise certain clinical skills. Difficulties with accessibility of the CSL for guided practice and self-directed learning because of the limited spaces available. This negatively impacted the opportunity for students to improve their clinical skills.

Aspects of the organisational environment need improvement with regard to human resources for CSL activities to ensure quality clinical teaching and learning opportunities.

Within the psychosocial environment of the CSL, the expectation of feedback provision during clinical sessions was identified. Nursing students reported on support provision from the clinical facilitators that reflected positively on their learning in the clinical environment.
